# Work ethics and its relationship with workplace ostracism and counterproductive work behaviours among nurses: a structural equation model

**DOI:** 10.1186/s12912-024-01785-4

**Published:** 2024-02-17

**Authors:** Nancy Sabry Elliethey, Ebtsam Aly Abou Hashish, Nariman Ahmed Mohamed Elbassal

**Affiliations:** Present Address: Faculty of Nursing, Alexandria University, Alexandria, Egypt

**Keywords:** Work ethics, Counterproductive work behavior, Work ostracism, Nurses, Structural equation model

## Abstract

**Background:**

The organization’s work ethics is the cornerstone to promoting positive nurses’ behaviours and overcoming counterproductive ones.

**Purpose:**

The current study aims to explore the relationship between work ethics (WEs) and counterproductive work behaviours (CWB) among nurses and testify to the mediating role of workplace ostracism (WO) in this relationship.

**Methods:**

A descriptive correlational study was conducted in an Egyptian hospital. A convenient sample of staff nurses (*N* = 369) who agreed to participate in the study answered work ethics, counterproductive work behaviours, and workplace ostracism questionnaires, which were proven to be valid and reliable study measures. Descriptive and inferential statistics were applied, and relationships were presented using structural equation modelling.

**Ethical Considerations:**

Ethics Committee approval, written informed consent, data privacy and confidentiality, and participants’ rights to voluntary participation and withdrawal were maintained.

**Results:**

The majority of nurses (78.5%) perceived a high level of work ethics while majority of nurses reporting low levels of counterproductive work behaviours and workplace ostracism (82.25%, 75.75%), respectively. In addition to the negative correlations, the findings revealed that WEs have a significant negative influence on each of CWB (β − 0.482, *p* < 0.005) and WO (β − 0.044, *p* < 0.005). The regression analysis showed that WEs can negatively predict about 15% of the variance in each of CWB and WO. On the other hand, WO has a positive effect on CWB (β 0.035, *p* < 0.021) and mediates the relationship between WEs and CWB.

**Discussion:**

Ostracism negatively affects the attitudes of nurses, which in turn results in negative behavioural outcomes (i.e., deviant behaviour).

**Conclusion:**

It is imperative for the hospital and nurse managers to establish a work environment that fosters support and cultivate work ethics and ethical work climate with the aim of managing negative work behaviours, enhancing nurses’ retention and satisfaction, and eventually improving the quality of patient care.

**Supplementary Information:**

The online version contains supplementary material available at 10.1186/s12912-024-01785-4.

## Background

Nurses must be equipped with the right values and work behaviours considering the importance of the organization’s strategic direction and financial capital to support the implementation of the vision and mission. Work ethics of Nurses are given priority because they can affect the effectiveness and performance of the organization. There is a great deal of debate on challenges relating to work ethics [1]. Employees who work for organizations that place a heavy emphasis on creating strong work ethics relevant to the difficulties they encounter will act ethically. Hence, work ethics have been demonstrated to significantly impact nurses’ behaviours. Nurses’ behaviour in an organization is divided into two parts: positive and negative behaviours [2]. Positive behaviour refers to an attribute that is associated with favourable outcomes resulting from an organization’s achievements, such as enhanced work productivity and superior performance. Negative behaviour is related to factors that affect the achievement of an organization’s goals [2], such as counterproductive work behaviour (CWB), which is a type of aggressive behaviour [3]. Work Ostracism (WO) is another antecedent of CWB; when nurses are ostracized by their colleagues, they begin to feel helpless, depressed, alienated, and unworthy, which in turn causes them to engage in counterproductive work behaviours (CWB) [4, 5]. Nurses’ awareness and practice of work ethics are crucial for their behaviours, ideas, and assigned obligations [1]. Hence, this study aims to extend nursing research to the relationship between work ethics and nurses’ work behaviours.

### Literature review

#### Work ethics (WEs)

Work ethics (WEs) is important in today’s health care settings; it is the key to the ethical behaviour of nurses [6]. WEs refers to a moral concept and value system that serve as a normative rule for how employees should conduct themselves while working for the organization [7, 8]. Furthermore, it is defined as a learned, religious-free, multidimensional concept reflected in behaviours, comprising attitudes and beliefs related to work but not a job [9]. WEs have the power to either motivate or dissuade people from committing crimes [5]. Putting work ethics into practice can considerably increase performance by using a variety of tactics to develop an ethical culture among the workforces [10].

Panigrahi and Al-Nashash [10] proposed that work ethics is a multidimensional concept that could be measured with different elements. Care, ethics code, regulations, instrumental, independence, hard work, and work as goal and time utilization are just a few of WEs element. The concept of work ethics is widely recognized as a fundamental soft skill that may significantly shape individuals’ attitudes and behaviours within the workplace [9, 11]. It plays a pivotal role in determining key organizational behaviours, including job performance, work quality, productivity, and organizational citizenship. The acquisition of work ethics is a process of skill development that individuals undergo throughout their lifetime, wherein they observe and emulate others before formulating their own views and behaviours. The development of work ethics is believed to be a gradual process influenced by personal experiences and the guidance of significant individuals such as educators, parents, peers, supervisors, and notable figures. The manifestation of learning can be observed through external work behaviours, work attitudes, and work habits [12, 13]. Research has revealed that adherence to work ethics has the potential to mitigate and regulate both deviant and ineffective work behaviours [14].

### Counterproductive work behaviours (CWB)

One of the most common issues at work is deviant conduct [15–17]. Sabotage, hostility, and physical or verbal abuse are examples of behaviours that primarily violate moral and ethical standards [18, 19]. These actions are all included in the broader category of “counterproductive job practises” or counterproductive work behaviours (CWB) [20, 21]. CWB reflects intentional, incorrect behaviours that have the potential to hurt both an organization and its employees [22]. CWB encompasses a broad range of aggressive or unfavourable organizational behaviours, such as production deviance, retreat, offending co-workers, delivering inferior work, and doing subpar work. It should be mentioned that these detrimental organizational practices directly affect both people and organizations in negative ways. These effects include negative social, monetary, and psychological effects like decreased productivity, dedication, loyalty, and job satisfaction. These effects also include a rise in employee absenteeism and turnover rates [23].

CWB has been classified into two types: actions directed towards organizations (CWB-O) and those directed towards individuals (CWB-I) by taking overly extended breaks, feigning illness, and staying at home from work, or, in some cases, signing a colleague’s presence in the office. Spreading untrue rumours about others, bullying, employing force, and verbally or physically abusing people are all examples of CWB-I [24]. In addition, CWB may be the outcome of the nurses’ facing ostracism in the workplace that makes them feel powerless, unhappiness, hostility, and unworthiness [25].

### Workplace ostracism (WO)

Workplace ostracism (WO) is defined as the degree to which people perceive a feeling of being ignored or left out by other colleagues [26]. In the workplace, WO takes different forms of exclusion, such as avoiding eye contact, leaving the room when someone enters, or moving someone to a faraway place [2, 27]. WO has been found to have negative effects on various aspects of employee well-being and organizational functioning. Specifically, it has been associated with increased levels of job anxiety, less inventive work behaviour, reduced efficiency, and diminished organizational performance. Moreover, it is worth noting that WO also contributes to an augmented emotional load, as it induces heightened levels of stress and emotional fatigue. Consequently, these factors give rise to unfavourable work-related attitudes, including job dissatisfaction, a dearth of emotional attachment, and a propensity to consider leaving the organization [28]. Nurses who are ostracised have a painful experience that prevents them from fulfilling their fundamental social needs of self-esteem, meaningful existence, belongingness, and control [29]. Ostracism is a prevalent phenomenon amongst nursing professionals in public sector hospitals who always need quality interaction to perform their jobs effectively. Therefore, when they are ostracised by their colleagues, these nurses start to feel helplessness, dejection, alienation, and unworthiness, which ultimately lead to CWB [4].

### Underpinning model

The General Aggression Model (GAM) developed by Allen, Anderson & Bushman [30] is a thorough, all-encompassing foundation for comprehending aggression. It examines how social, cognitive, psychological, developmental, and biological factors affect behavioural outcomes such as aggression or nonaggression [31]. According to GAM, both individual and environmental factors contribute to the development of aggressive behaviours [31]. Aggressive behaviour, which includes counterproductive work actions, is influenced by both individual and situational factors [32]. One of the most common negative situations in interpersonal communication is being ostracised, and the type and specific circumstances at the time of occurrence may have a greater impact on the subsequent internal state and behavioural outcomes than would otherwise be the case due to the relative stability of personal factors, situations, and especially ostracism [33, 34]. Given the framework of the General Aggression Model (GAM), our hypotheses posit a correlation between workplace supportive elements, specifically work ethics, and the incidence of negative or aggressive work behaviours.

### Research hypotheses

**Fig. 1:**
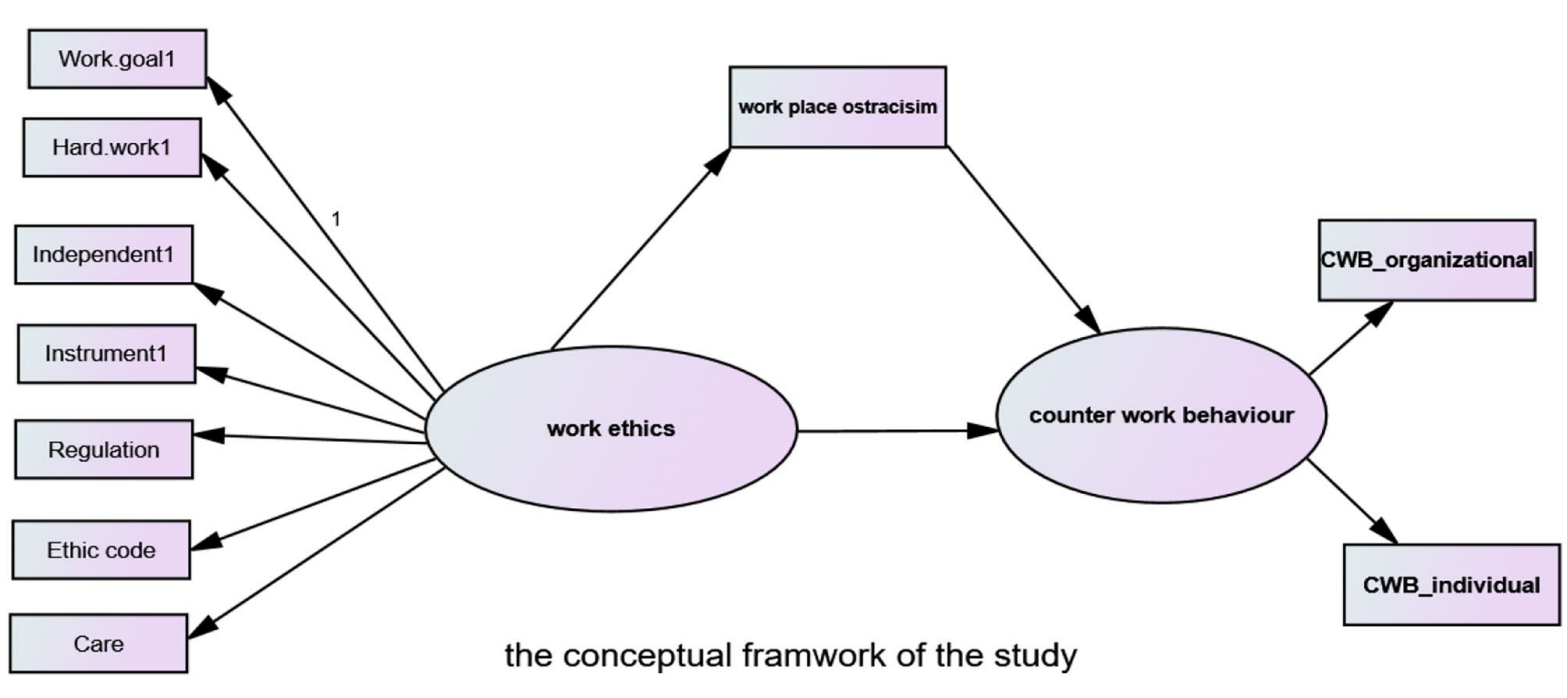
Proposed conceptual fremework

From the previous conceptualizations, we proposed a conceptual model for this study (Fig. 1). Given that work ethics is the independent variable, counterproductive work behaviour is the dependent variable, and workplace ostracism plays a mediating role, the following hypotheses are postulated:

H_1_: There is a significant correlation between workplace ethics, workplace ostracism, and CWB among nurses.

H_2_: Workplace ostracism can play a mediating role in the relationship between workplace ethics and nurses’ CWB.

### Significance of the study

Hitlan and Noel [35] identified that workplace exclusion, ostracism, and personality contribute to CWB, and higher degrees of organizational CWB were associated with higher levels of supervisor exclusion. Additionally, there was a strong correlation between interpersonal CWB and co-worker exclusion. A person’s ethical issues are based on their relationships and commitments in the workplace, where proper conduct isn’t always obvious and there aren’t any set rules to follow. Ethics problems develop when supervisors or colleagues hold opposing views on topics like strategy, aims, policy, and management. Conflicts of interest, relationships inside the organization, honesty, integrity, and communication can all be used to describe ethical issues [34].

A strong work ethic supports one’s desires and aspirations and is associated with one’s initiative to achieve the objectives of nurses and their organization. Lack of productivity, dependability, responsibility, and a growing circle of unprofessional or unhealthy connections are all symptoms of a bad work ethics and could be motives for CWB [34]. So, it’s important to search in these areas, as past research has left a gap to investigate the relationship between work ethics and counterproductive work behaviour and workplace ostracism as a mediating role.

### Aim of the study

The study’s aim was to investigate the relationship between work ethics and counterproductive work behaviour, with workplace ostracism playing a mediating role.

## Methods

### Design and setting

A descriptive correlational research design was conducted in an Egyptian hospital.

### Participants and sample

The study focused on nurses employed in the inpatient care unit of the hospital as the target population. To identify eligible nurses who satisfied the specified inclusion criteria, a convenience sampling method was employed. The study included a cohort of nurses who willingly volunteered to take part and had accumulated a minimum of three to six months of experience in their respective clinical practice. We excluded newly employed nurses, and interns because of potential limitations to their complete familiarity and engagement with the work environment. The sample size was determined using the Raosoft sample size calculator, based on the specified parameters. The population size was recorded as 660 individuals, with a margin of error of 5 units. The significance threshold, denoted as p, was set at *p* ≤ 0.05. Therefore, a minimum sample size of 244 is recommended. A total of 400 nurses were administered a questionnaire in order to obtain a suitable sample size. Among the participants, a total of 369 nurses successfully completed the surveys and subsequently returned them, thereby constituting the designated target sample.

### Instruments and measurements

Three standardized questionnaires were used in this empirical study, namely the work ethics multidimensional questionnaire, the counterproductive work behaviours scale, and the workplace ostracism scale:

### Work ethics (WEs) questionnaire

Panigrahi and Al-Nashash [10] developed the multidimensional work ethics questionnaire. It consists of 25 items measured in 7 dimensions: care (3 items such as; I take care of people inside and outside organization), ethics code (4 items such as I make decision based on professional code of ethics), regulation (4 items as, I obey rules set for employee working attitude), instrument (4 items as, I am instrumental among people within the organization), independent (3 items as, I consider moral beliefs based on principles), hard work (3 items as, I am always willing to work), and work goal (4 items as, I am always self-esteemed with the work I do). Responses on work ethics employed a 5-point Likert scale, with (1) indicating less important and (5) indicating high important.

### Counterproductive work behavior (CWB) scale

The Counterproductive Work Behaviour (CWB) Scale was originally developed by Dalal et al. [36]. It consists of 23 items measured in two dimensions: CWB-I is directed at individuals (10 items such as, verbally abusing someone at work) and CWB-O is directed at the organization (13 items such as, you stole something belonging to your employer). Participants are asked to rate each statement based on how often they do the listed behaviours on a five-point Likert scale (1 = *never* to 5 = *always*). Higher scores indicate a higher frequency of employees engaging in counterproductive work behaviours.

### Workplace ostracism (WO) scale

The Workplace Ostracism (WO) Scale was developed by Ferris et al. [26]. and consists of 10 items. Participants are asked to rate each statement on a five-point Likert scale (1 = *never* to 5 = *always*), for example, “others ignored you at work,” based on how often they feel the sentiment. Higher scores indicate a stronger sense of workplace ostracism.

### Translation, validity, and reliability

Initially, a comprehensive translation of all tools was undertaken to ensure their compatibility with the Arabic language and alignment with Egyptian culture, as well as their appropriateness for various nurses’ educational levels. Subsequently, a panel of five academic experts rigorously assessed the content validity and linguistic fluency of the translated tools. The experts were asked individually to assess the instrument’s qualities in terms of item relevancy, comprehensiveness, and comprehension. In order to ensure accuracy and reduce potential threats to the study’s validity, a few items were adjusted for greater clarity before being back-translated into English by linguists. Based on their agreement rating, the resulting content validity index (CVI) for the WEs instrument was 88.99, for the CWB instrument it was 89.13, and for the WO instrument it was 86.2. These findings provide evidence that these instruments possess validity. Also, they approved the back-translation of the tools. After that and before the final administration of the tools, a pilot study involving 30 nurses was carried out to assess the tools’ clarity and applicability as well as to determine how long it would take them to complete the study questionnaires. Additionally, the internal reliability of the study tools was evaluated using Cronbach’s alpha correlation coefficient. The findings demonstrated the validity of the instruments, with correlational coefficients for the WEs, CWB, and WO of 0.876, 0.950, and 0.94, respectively, while the statistical significance level was *p* ≤ 0.05.

### Data collection

The administrative authority in the designated setting gave written consent for the required data collection. The researchers first met with the nurse managers of the units to obtain a sampling list of all staff members with their years of experience and obtain their consent to interview the nurses in accordance with their schedules and break times. The nurses who consented to take part in the study were then Individually given the questionnaires by the researchers after being fully briefed at the agreed-upon time with the required instructions. Between November 2022 and February 2023, data were gathered from nurses after gaining their consent via the questionnaires.

### Ethical considerations

The study received approval from the Research Ethics Committee of the Faculty of Nursing, Alexandria University (approval number: IRB00013620(9/19/20). All procedures were conducted in accordance with ethical guidelines for human research, adhering to applicable regulations. Additionally, approval to collect data was secured from the hospital’s managers. Participants provided written informed consent after a comprehensive explanation of the study’s objectives, potential risks and benefits, and their voluntary right to withdraw. The researchers prioritized data privacy, confidentiality, and participant anonymity.

### Data analysis

Item response theory (IRT) and confirmatory factor analysis (CFA) analysis were performed using AMOS version 24 after all data had been entered and validated for missing information using SPSS software version 24. To evaluate the internal reliability of the study’s tools, Cronbach’s alpha correlation coefficient was used. Demographic and professional traits were described using frequency and percentages. To quantify the variables under research, the arithmetic mean, and standard deviation (SD) were utilised as measures of central tendency and dispersion, respectively. To investigate variations in perceived values and a few demographic factors, an independent t-test and one-way analysis of variance (ANOVA) were computed. The nature of the association between the variables was analyzed using the Pearson correlation coefficient (r). A Pearson correlation coefficient of 0.1 suggests a weak relationship, 0.3 indicates a moderate relationship, and 0.5 suggests a strong relationship. The statistical significance of these relationships was assessed using an alpha level of p *≤* 0.05.

The validity and reliability of each construct were examined using an exploratory factor analysis (see Supplementary Fig. 1). Convergent validity is attained with factor loadings of _0.50, average variance extracted (AVE) of _0.50, and composite reliability (CR) of _0.70 since the latent constructs of the hypothesised framework are reflective. In the meantime, discriminant validity is assessed by contrasting the AVEs’ square roots with the relevant factor’s correlations. Weak factor loadings (0.50) on items prevented them from being included in the study.

## Results

### Nurses’ characteristics

According to Tables 1 and 78.6% of nurses were female, and 59.3% of them were between the ages of 20 and 30. Only 2.7% of nurses were over the age of 50. The majority (59.4%) received a nursing diploma from a secondary technical nursing school (36.9%) or a technical health institute (22.4%), while the remaining 40.7% earned a bachelor’s degree in nursing science. 78.6% of the included nurses had between one and ten years of experience, and 81.3% worked in medical-surgical units.

**Table 1:** Distribution of studied nurses according to their demographic and professional characteristics (*N* = 369)

Sociodemographic data	Frequency	%
**Age**		
20 years to 30	219	59.3
31–40 years	100	27.1
40–50years	40	10.8
More than 50 years	10	2.7
Min.– Max.	12–65	
Mean ± SD.	23.07 ± 6.41	
**Sex**		
Male	79	21.4
Female	290	78.6
**Educational preparation degree**		
Bachelor	150	40.7
Practical (school diploma)	136	36.9
Technical (diploma of higher technical institute)	83	22.4
**Working unit**		
ICU	69	18.7
Medical -Surgical	300	81.3
**Years of nursing experience**		
3 months-Less than year	20	5.4
1–10 years	290	78.6
11–20 years	50	13.6
21–30 years	9	2.4

Notes: SD: standard deviation

### Perceived level of work ethics (WEs), counterproductive work behaviours (CWB), and work ostracism (WO) among nurses

The majority of nurses (78.59%) perceived high WEs, as is evident from Table 2, and the overall mean percent score of WEs as perceived by nurses was 77.70%. Additionally, nurses reported high levels of overall WEs in their workplace based on the average of all WEs dimensions, including care, ethics code, regulation, instrument, independent, hard work, and goal aspects. The majority of nurses (86.45%) had a negative opinion of CWB; however, they thought CWB-O directed at the organization was slightly worse than CWB-I directed at specific individuals (39.69%). Additionally, with a mean percent score of 41.74%, the majority of nurses (82.11%) reported having a negative opinion of WO.

**Table 2:** Levels and Mean score of work ethics, CWB, and WO among studied nurses

Variables	Level	Frequency	%	Mean ± Sd	Mean score %
**WEs**	Low	14	3.79	120.44 ± 13.13	77.70%
	Moderate	65	17.62		
	High	290	78.59		
Care	Low	5	1.36	12.07 ± 1.80	80.47%
	Moderate	45	12.20		
	High	319	86.45		
Ethic code	Low	4	1.08	15.69 ± 2.18	78.45%
	Moderate	80	21.68		
	High	285	77.24		
Regulation	Low	4	1.08	16.21 ± 2.24	81.05%
	Moderate	55	14.91		
	High	310	84.01		
Instrument	Low	7	1.90	14.29 ± 2.38	71.45%
	Moderate	135	36.59		
	High	227	61.52		
Independent	Low	5	1.36	11.39 ± 1.82	75.93%
	Moderate	99	26.83		
	High	265	71.82		
Hard work	Low	5	1.36	12.11 ± 1.91	80.73%
	Moderate	50	13.55		
	High	314	85.09		
Work goal	Low	9	2.44	15.47 ± 2.48	77.35%
	Moderate	85	23.04		
	High	275	74.53		
**CWB**	Low	319	86.45	45.13 ± 14.85	39.24%
	Moderate	45	12.20		
	High	5	1.36		
CWB-O	Low	310	84.01	25.80 ± 8.34	39.69%
	Moderate	55	14.91		
	High	4	1.08		
CWB–I	Low	310	84.01	19.33 ± 6.56	38.66%
	Moderate	49	13.28		
	High	10	2.71		
**WO**	Low	303	82.11	20.87 ± 6.58	41.74%
	Moderate	55	14.91		
	High	11	2.98		

Notes: SD: standard deviation

### Relationship among WEs, CWB, and WO

**Fig. 2:**
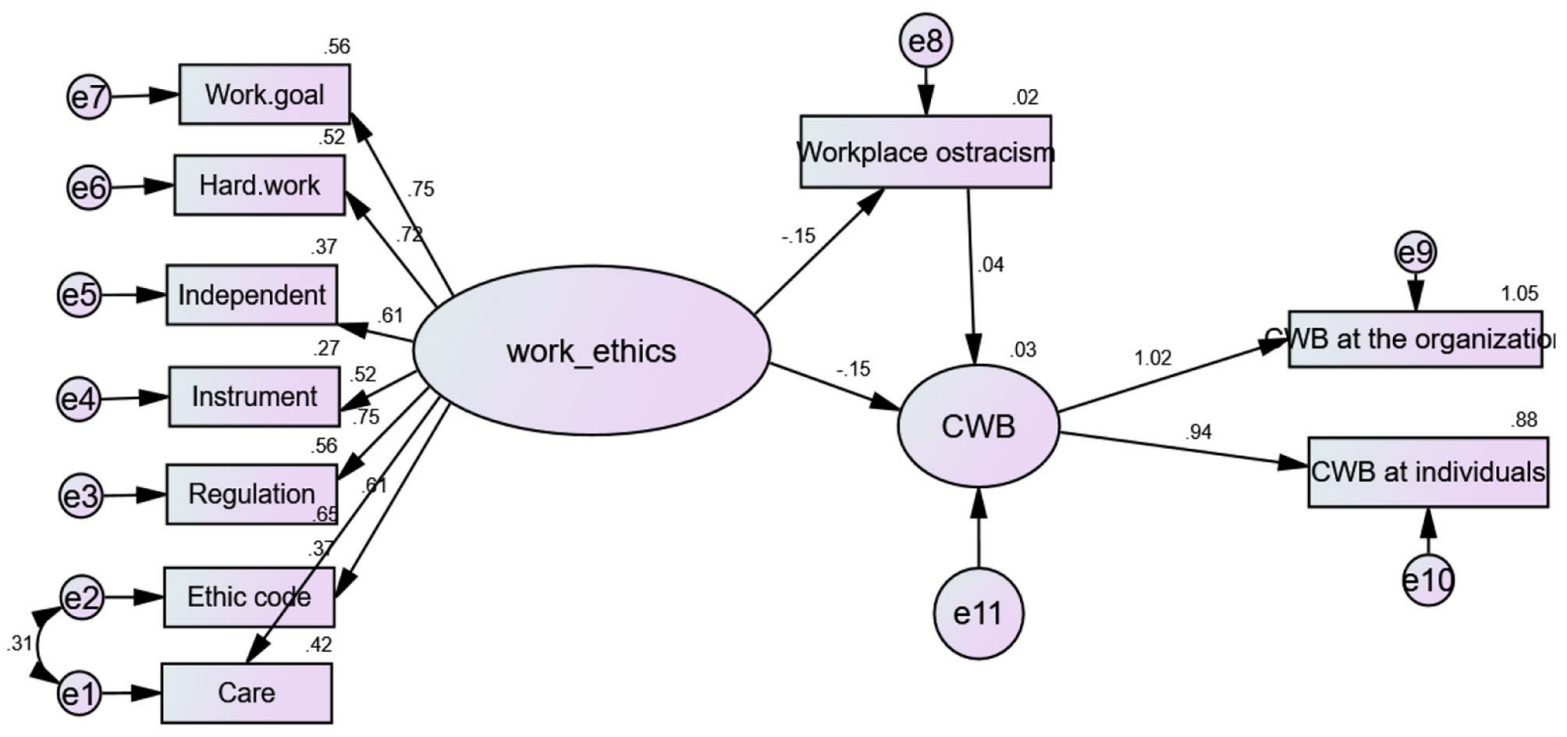
Standardized coefficient among WEs, CWB and WO with the mediating role of WO

In addition to the negative association between WEs and CWB, WO, and each other, Table 3 showed that WEs significantly influences CWB (β − 0.482, p 0.005) and WO (β − 0.044, p 0.005) adversely, with a regression coefficient of − 0.151 and − 0.149, respectively. This suggests that WEs can predict roughly 15% of the variance in each of CWB and WO negatively. Contrarily, WO supports Hypothesis 1 by having a favourable impact on CWB (β 0.035, p 0.021) and being able to predict roughly 3.8% of the variance in CWB. Additional data corroborate Hypothesis 2 by demonstrating that WEs significantly affect CWB via WO (β-0.482, p 0.01). Figure 2 shows that WO partially mediates the relationship between WE practice and CWB. More values are illustrated in supplementary Table 2.

**Table 3:** Standardized regression coefficient weights among WE, CWB and WO with the mediating role of WO

Variables			Beta	R^2^	S.E.	C.R.	P
Work ethics^a^	<---	Work Ostracism	− 0.044	− 0.151	0.016	-2.707	0.005*
Counterproductive Work Behaviour^b^	<---	Work ethics	− 0.482	− 0.149	0.179	-2.691	0.005*
Counterproductive Work Behaviour^c^	<---	Work Ostracism	0.035	0.038	0.048	0.737	0.021*

Note. a(*r*=-0. 127, *p* = 0.01), b(*r*=-0.141, *p* = 0.001), c(*r* = 0.114, *p* = 0.001). Model fit parameters RFI, NFI; IFI; RMSEA.806.853.973.032 respectively, r = Pearson correlation; R^2^ = regression coefficient; CFI = Comparative fit index; and RMSEA = Root Mean Square Error of Approximation. * p significant ≤ 0.05

### Work ethics, counterproductive work behaviour, workplace ostracism, and nurses’ demographics

With the exception of nurses’ sex and educational background, Table 4 did not reveal any statistically significant differences in the perceived factors according to nurses’ demographic and professional characteristics. Compared to female nurses, male nurses had higher mean values for CWB and WO (t = 2.361(0.019); 3.715(0.000, respectively). A higher mean of WO was also seen among technical degree nurses compared to baccalaureate nurses.

**Table 4:** Perceived WEs, CWB, and WO according to nurses’ professional and demographic characteristics

Sociodemographic data	WEs	CWB	WO
	Mean ± SD.	Mean ± SD.	Mean ± SD.
**Age**			
20 years or less	119.87 ± 14.52	45.66 ± 15.07	21.35 ± 7.02
21–30 years	121.53 ± 10.58	43.56 ± 15.38	19.89 ± 6.37
31–40 years	120.45 ± 11.69	44.86 ± 13.12	20.49 ± 4.86
More than 40 years	123.10 ± 7.17	49.60 ± 11.74	21.30 ± 3.83
Test of sig.(p)	F = 0.521(0.668)	F = 0.789(0.500)	F = 1.242(0.294)
**Sex**			
Male	118.96 ± 16.59	47.97 ± 17.51	22.83 ± 7.31
Female	121.00 ± 11.56	44.06 ± 13.60	20.13 ± 6.13
Test of sig.(p)	t = 1.380(0.168)	t = 2.361(0.019)	t = 3.715(0.000) *
**Educational preparation**			
Bachelor	117.33 ± 13.14	44.20 ± 17.19	16.80 ± 5.23
Practical	121.19 ± 11.59	45.79 ± 15.84	20.63 ± 6.59
Technical	120.22 ± 13.91	44.82 ± 14.18	21.25 ± 6.57
Test of sig.(p)	F = 0.676(0.509)	F = 0.221(0.802)	F = 3.422(0.034) *
**Working Unit**			
ICU	119.99 ± 13.77	44.92 ± 15.42	21.04 ± 6.69
Medical–Surgical	122.54 ± 9.40	46.06 ± 11.87	20.10 ± 5.99
Test of sig.(p)	t = 1.483(0.139)	t = 0.582(0.561)	t = 1.090(0.276)
**Years of experience**			
3 months-Less than year	121.95 ± 11.35	38.90 ± 9.05	22.35 ± 6.46
1–10 years	120.10 ± 13.77	45.53 ± 15.52	20.73 ± 6.91
11–20 years	121.61 ± 10.77	45.21 ± 12.92	21.03 ± 4.92
21–30 years	125.33 ± 4.97	45.83 ± 12.51	18.33 ± 3.01
Test of sig.(p)	F = 0.588(0.623)	F = 1.251(0.291)	F = 0.685(0.561)

t: independent samples test, F: one-way ANOVA, * p significant ≤ 0.05

## Discussion

Maintaining good work ethics and its positive effect on nurses’ behaviours is one of the incredible problems confronting healthcare organizations [37, 38]. This correlational and descriptive study expanded previous research regarding the impact of work ethics on counterproductive behaviours when mediated by workplace ostracism. The finding reveals that work ethics (WEs) was negatively correlated with each of counterproductive behaviours (CWB) and work ostracism (WO) and could negatively predict about 15% of their variance. Also, work ostracism was partially mediated with WEs and CWB and has had a positive effect on CWB with 50%, which supports Hypotheses 1 and 2. These results implied that the higher the work ethics, the lower the perceived level of both CWB and WO, and any increase in WO can lead to more CWB among nurses. This perspective could be supported by the descriptive levels and mean scores of the studied variables, which showed a higher perception of WEs and a lower perception of CWB and WO. This result might represent that the hospital under study has a supportive work ethics standard that clearly define the required actions, positive and negative behaviour, published and oriented work standards, and a high level of accountability. It can be deduced that the cultivation of strong work ethics and the establishment of a supportive work environment would likely discourage nurses from engaging in counterproductive and ostracising behaviours. Conversely, the presence of ostracism within an organizational context is expected to result in the manifestation of counterproductive behaviour. Consequently, the present research hypotheses have been validated. This justification concurs what is presented by Abou Hashish [37].

Similar to our findings, Abdullah and Halim [39] showed that individuals with strong work ethics are less likely to engage in CWB, whereas individuals with a low work ethic are more likely to engage in CWB. Howard et al. [40] and Budiman et al. [41] stated that work ethics is the most important element in influencing and preventing counterproductive work practices. De Wolde et al. [42] reported that work ethic is also an important element in organizational development and contributes to a less CWB. Therefore, hospital and nurse managers should provide appropriate ethic codes and guidelines related to professional work ethics as well as an intervention on behaviour change towards integrity. Moreover, and concurs with our findings Ebrahimi et al. [25] reported high positive correlation between CWB and WO. Therefore, when nurses ostracised by their colleagues, start to feel powerlessness, unhappiness, hostility, and unworthiness, which ultimately cause counterproductive work behaviours. Ostracism caused employees’ psychological burden as emotional exhaustion, work tense and these pressures could upset their work and cause work conflict [43, 44].

Also, Shafique et al. [45] showed that WO positively influences negative attitudes and behaviours at the workplace, such as job tension and different behaviour, and negatively affects positive workplace attitudes and behaviours. Likewise, Chung & Yang [46], Yang & Treadway [47], and Afsheen [48] confirmed that WO increased counterproductive behaviours. In this respect, Chung [49] reported that WO is a stressor, and the provision of psychological empowerment can mitigate the negative effect of ostracism on behavioural outcomes.

Regarding the descriptive level of the studied variables, the present study revealed a high level of work ethics and its related dimensions, including care, ethics code, regulation, instrument, independent, hard work, hard goal dimensions, with the highest mean of work ethics related to regulation, care, and hard work dimensions. Parallel with this high work ethics, nurses reported lower level of CWB and its related dimensions and work ostracism. This result might be attributed to the presence of a positive and professional value system focusing on the moral values of work, self-discipline, individual responsibility, and the importance of self-reliance, ethical guideline, trust relationship between managers and nurses, and collaborative conflict management. In addition to the possibility and tendency of nurses to please their managers and to be perceived as committed and engaged to their work regulations. The current results agree with Ali and Johl [8] who revealed that nurses are more likely to react with CWB at a low level. Also, Yao Jr [50] stated that around two-thirds of studied subjects had low levels of counterproductive work behaviour.

In addition, Zahid et al. [51] stated that the studied nurses have a low level of WO. Similarly, Chen and Li [52] and Armstrong [53] found that more than half of the staff nurses had a low level of WO, while a minority of them had a high level of workplace ostracism. In contrast, Mlika et al. [54] indicated that ostracism is frequently observed in healthcare organizations. Ebrahim and Eldeep [43] reported that two-thirds of the studied nurses had a moderate level of workplace ostracism and one fifth had low workplace ostracism. They suggested that bias from management, peer jealousy, and actual health problems among nurses were at blame. Also, Xia et al. [55] reported that 66% of employees have suffered from ostracism at the workplace. Moreover, El-Guindy et al. [56] illustrated a moderate level of workplace ostracism among staff nurses. Potential causes of such a high WO include poor performance, a toxic workplace, inadequate resources, and a shortage of qualified workers. According to Howard et al. (41), leadership traits have been identified as the most influential factors related with workplace ostracism. In this regard, Zhou et al. [44] emphasized the role of a supportive leader who practices work ethics and promotes the quality of work behaviours and relationship among nurses. Spector and Fox [57] found that professional work ethics are learned from the surroundings and work environment planning. When combined with each health service provider’s awareness, this professional work ethic can improve leader-follower relationships and eliminate CWB. Also, Qin et al. [58] suggested that workers who practice work ethics tend to have a higher quality of relationship and are more likely to mitigate CWB at work.

What is more, in the current study, nurses’ demographics and professional characteristics reported no significant effect on the perceived variables except for nurses’ sex and educational qualifications with perceived WO and CWB. Male nurses showed a higher mean of CWB and WO than female nurses. Also, technical degree nurses showed a higher mean of WO than baccalaureate nurses. This result might be related to the personality differences between male and female nurses in responding to and managing negative work behaviours, in addition to the experience and educational preparation of bachelor nurses. Growing in nursing experience and educational level helps nurses decrease counterproductive behaviour. In this vein, many researchers presented the effect of nurses’ variables on their work behaviours. For instance, Ebrahimi et al. [25] reported statistically significant relationships between ostracism and employment status, nurses’ education, and current physical disorders. Ugwu et al. [59] presented a significant correlation between gender and counterproductive behaviour, yet no correlation with nurses’ age. Sarwar et al. [60] detected a high and significant correlation between age and workplace ostracism.

Meanwhile, Lawal et al. [61] demonstrated that no gender difference was found in CWB among supporting staff and age, pay satisfaction, and intent to leave significantly predicted counterproductive work behaviour with age. While acknowledging the potential influence of nurses’ unique and personal aspects on their perspective, researchers should explore this aspect in future studies. Therefore, assessment of work ethics and negative work behaviours as CWB and WO should consider the interaction between organizational and individual factors, which could provide a better understanding of group dynamics and individual attitudes towards the work environment and its culture.

### Strengths and limitations

The present study’s results have the potential to expand the existing body of nursing literature and contribute to the fields of nursing and hospital management. Specifically, these findings shed light on the influence of work ethics on shaping nurses’ work behaviours, leading to a decrease in counterproductive work behaviours and work ostracism. Nevertheless, it is important to acknowledge the limitations of this study. The data was obtained using the self-report methodology, which may include inherent biases and limit the generalizability of the results [62]. The study examined nurses’ perceptions of work ethics and negative work behaviours, and nurses might consider their fear and desire to please their managers. Respondents may engage in the phenomenon known as “faking good” due to the presence of social desirability bias when they deliberately alter their responses to conform to societal expectations and avoid potential criticism. This perspective has the potential to introduce bias in the responses of the participants, consequently influencing the true relationships that exist between the variables under investigation. Certain future studies are recommended in the following section.

### Conclusion and recommendation

The current study makes several contributions to the understanding of healthcare organizational dynamics and nurses’ behaviours. This study aims to examine the effect of work ethics on the occurrence of workplace ostracism (WO) and counterproductive work behaviour (CWB). Additionally, it explores work ostracism as a precursor to CWB. The findings indicate a substantial negative correlation between nurses’ strong sense of work ethics and their engagement in unproductive work behaviours. This relationship has predictive value and suggests that a higher perception of work ethics can directly decrease such behaviours. Additionally, work ethics can also indirectly reduce counterproductive work behaviours by mitigating work ostracism among nurses. Hence, work ethics serve as a catalyst, mitigating the negative impact of workplace ostracism (WO) and counterproductive work behaviour (CWB) on nurses.

In summary, maintaining Ethical values and standards in the workplace is paramount within contemporary management since the conduct of employees profoundly influences an organization’s ability to thrive in the context of global competitiveness. In order to effectively execute their routine responsibilities, every organization necessitates a robust understanding of workplace ethics.

### Implications of the study

The findings of this study have significant implications for healthcare and nursing institutions and future research.

### Implications to practice

Hospital and nurse managers are responsible for establishing and sustaining conducive work cultures that foster strong work ethics and mitigate instances of unacceptable workplace behaviours. Provision of the required care standard, adherence to the ethics code, communicating and maintaining regulation, promoting independence and autonomy, and hard work aligned organizational goals are essential elements for cultivating the positive effect of work ethics.

Despite being observed at low levels, both CWB and WO persist and possess the capacity to escalate if appropriate measures are not taken to minimise and prevent their occurrence. Hence, it is imperative for nurse managers to offer assistance and guidance to nurses who have encountered or been subjected to such behaviours to mitigate any negative consequences for their job performance and overall job satisfaction. In order to enhance nurses’ understanding of counterproductive behaviours and workplace ostracism, as well as their potential ramifications and corresponding mitigation strategies, it is imperative for hospital and nurse managers to foster educational approaches. These strategies can prove beneficial in the long term, safeguarding the overall well-being of the nursing profession.

The implementation of frequent awareness and training sessions conducted by workplace managers pertaining to positive work climate, organizational and individual value systems, team building, conflict management, resilience-building, and self-efficacy represents a potentially effective approach for mitigating and preventing disruptive behaviour within the workplace and increasing conscientiousness towards the ethical ramifications of employees’ behaviours.

### Implications for future studies

#### Due to the stated limitations of the study, the following future research was suggested

Future investigations should utilize a triangulation of data collection sources and methods, including nurses, nurse managers, and physicians, as well as qualitative and quantitative methods. Furthermore, the recruiting strategy included convenience sampling, which raises concerns about the generalizability of the findings to the broader nursing population. Nevertheless, it is imperative for future research attempts to extend beyond the scope of this study to substantiate the impact of work ethics on negative behaviours (namely, counterproductive work behaviours and workplace ostracism) within a broader context. This can be achieved by employing other data collection methods and examining diverse samples. Furthermore, the study specifically investigated the impact of work ethics alone on negative workplace behaviours. A more comprehensive understanding of the factors contributing to counterproductive workplace behaviours can be achieved by investigating the effects of various individual-related and organizational variables, such as personality characteristics and organizational support. Also, the effect of work ethics could be more evident through investigating its relationship with positive outcomes such as organization citizenship behaviour, job embeddedness, commitment, resilience, and work productivity.

### Electronic supplementary material

Below is the link to the electronic supplementary material.

Supplementary Material 1

## Data Availability

All data generated or analysed during this study are included in this published article [and its supplementary information files].

## Declarations

### Content of the manuscript

We confirm that the content of the manuscript has not been published or submitted for publication elsewhere.

### Approval confirmation

All authors have approved the manuscript for submission.

## Abbreviations

ANOVA: Analysis of variance
AVE: Average variance extracted
CFA: Confirmatory factor analysis
CR: Composite reliability
CVI: Content Validity Index
CWB: counterproductive work behaviours
CWB-O: counterproductive work behaviours directed towards organizations
CWB-I: counterproductive work behaviours directed towards individuals
GAM: General Aggression Model
WEs: Work Ethics
WO: Workplace Ostracism
IRT: Item response theory
SPSS: Statistical package of social science
SD: Standard deviation
