# Supplementary material for: Work ethics and its relationship with workplace ostracism and counterproductive work behaviours among nurses: a structural equation model
Source: BMC Nurs. 2024 Feb 17;23:126. doi: 10.1186/s12912-024-01785-4 (PMC10874021; doi:10.1186/s12912-024-01785-4)
Supplement: Supplementary file 1 — Supplementary Material 1 [file 12912_2024_1785_MOESM1_ESM.docx]

**Supplementary file:**

**Work Ethics and its Relationship with Workplace Ostracism and Counterproductive Work Behaviors among Nurses: A Structural Equation Model**

Supplementary Table 1. *Exploratory Factors loading*

| **Factors** | **Rotation sum squared loading** |
| --- | --- |
| 1 | 12.898 |
| 2 | 9.853 |
| 3 | 8.968 |
| 4 | 7.421 |
| 5 | 6.770 |
| 6 | 6.650 |
| 7 | 6.312 |

**
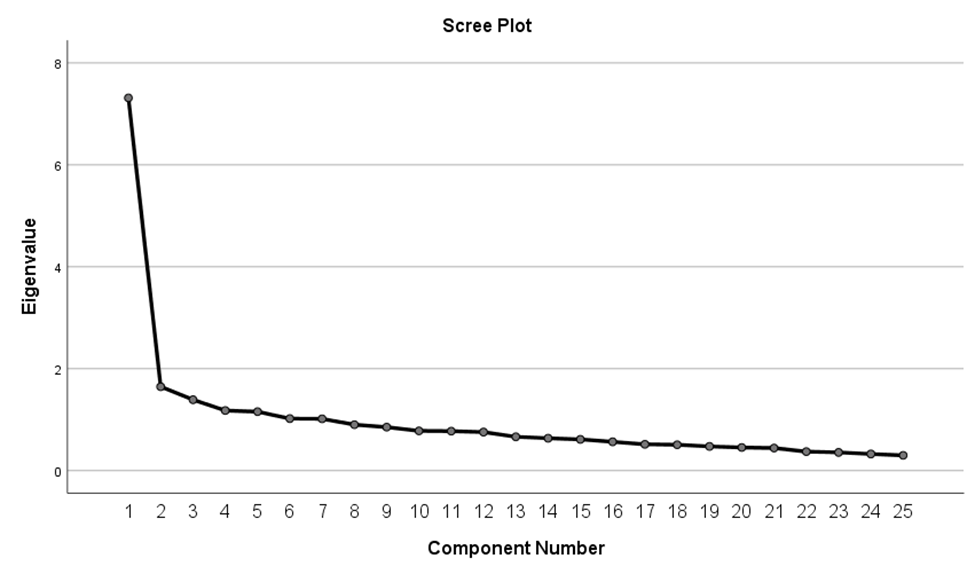
**

**Supplementary Figure 1: Exploratory Factor Analysis**

**Supplementary table 2.** *Standardized Regression Weights: (Group number 1 - Default model)*

| variables | | | Beta | R^2^ | S.E. | C.R. | P |
| --- | --- | --- | --- | --- | --- | --- | --- |
| Work_ethics^a^ | <--- | total.Ostracism | -.044 | -.151 | .016 | -2.707 | .005 |
| Counterproductive_Work_Behaviour^b^ | <--- | Work_ethics | -.482 | -.149 | .179 | -2.691 | .005 |
| Counterproductive_Work_Behaviour^c^ | <--- | total.Ostracism | .035 | .038 | .048 | .737 | .021 |
| Instrument1 | <--- | Work_ethics | .653 | .510 | .071 | 9.262 | *** |
| Regulation | <--- | Work_ethics | .911 | .745 | .067 | 13.657 | *** |
| Independent1 | <--- | Work_ethics | .596 | .602 | .054 | 10.988 | *** |
| Ethics code | <--- | Work_ethics | .771 | .654 | .064 | 11.961 | *** |
| Care1 | <--- | Work_ethics | .664 | .671 | .054 | 12.277 | *** |
| Hard work | <--- | Work_ethics | .746 | .716 | .057 | 13.118 | *** |
| Work goal | <--- | Work_ethics | 1.000 | .742 |  |  |  |
| CWB Directed at the organization | <--- | Counterproductive_Work_Behaviour | 1.000 | 1.014 |  |  |  |
| CWB directed at individuals | <--- | Counterproductive_Work_Behaviour | .738 | .947 | .076 | 9.756 | *** |

*Note. a(r=-0. 127, p= 0.01), b(r=-0.141, p= 0.001), c(r=0.114, p= 0.001). Model ﬁt parameters RFI, NFI; IFI; RMSEA.806.853.973.032 respectively*

*r = Pearson correlation; R^2^= regression coefficient; CFI = Comparative fit index; and RMSEA = Root Mean Square Error of Approximation.* ** p significant ≤ 0.05*
